# Music performance anxiety beyond coping: toward a creative arts perspective in higher music education

**DOI:** 10.3389/fpsyg.2026.1901672

**Published:** 2026-09-03

**Authors:** Jingwen Zhang

**Affiliations:** The University of Auckland, Auckland Central, New Zealand

**Keywords:** creative arts, educational approaches, higher music education, music performance anxiety, wellbeing

## Abstract

Music performance anxiety (MPA) remains a common challenge in higher music education, affecting performance quality, student confidence, and long-term wellbeing. While existing approaches have provided important tools for symptom reduction and coping, a primarily clinical or management-oriented focus may overlook how MPA is experienced within musical learning. This article develops a conceptual framework that brings together MPA research, higher music education scholarship, and creative arts-informed thinking, reframing MPA not only as a condition to be managed, but as an experience that can be explored through creative and reflective processes. The framework comprises four interrelated pedagogical processes: embodied awareness, expressive exploration, relational witnessing, and reflective meaning-making. By shifting the focus from management to meaningful engagement, the article contributes a pedagogical framework for rethinking MPA support in higher music education and establishes a conceptual foundation for future workshop development within students’ musical learning and wellbeing.

## Introduction

Music performance anxiety (MPA) is a common concern among musicians and has been widely examined in relation to its prevalence, risk factors, and treatment (Fernholz et al., 2019). Kenny (2011) conceptualizes MPA as a persistent form of anxious apprehension that involves cognitive, emotional, physiological, and behavioral symptoms. MPA is therefore more than ordinary nervousness before performance. It is a complex experience that can shape how musicians think, feel, prepare, perform, and evaluate themselves.

Within higher music education, this issue is particularly acute. Conservatoire and university environments are often characterized by repeated assessment, public performance, technical expectations, and pressure for artistic excellence. In this context, MPA should not be viewed only as a performance hurdle, but also as a wellbeing concern. Recent evidence from Rose et al. (2026) indicates that music students may score below general population norms in both physical and psychological health, Gilbert (2021) similarly reports that music majors show higher levels of anxiety and depression than non-music majors. These findings suggest that anxiety in music education is not merely an incidental individual problem, but part of a wider educational and institutional concern.

Although existing literature has made significant contributions to understanding MPA, it has often focused on coping mechanisms, clinical treatments, and symptom reduction, which tends to frame MPA primarily as a problem to be managed or controlled (Brugués, 2011). This emphasis may be insufficient for higher music education, where anxiety is closely connected to students’ embodied experience, relationships, artistic identity, and wellbeing. Therefore, this article develops a conceptual and educational perspective on MPA support that moves beyond treating anxiety only as something to suppress by emphasizing students’ embodied awareness, expressive exploration, relational witnessing, and reflective meaning-making.

The article first reviews psychological, pedagogical, and arts-informed discussions of MPA, before introducing a four-part conceptual framework. This contribution offering a conceptual basis for future workshop development. In this way, the article shows how a creative arts-informed model may extend existing MPA research toward more holistic and pedagogically grounded forms of support.

### Current progress in understanding and addressing MPA

#### Psychological and performance-based understandings of MPA

Research in music psychology and performance science has made an important contribution to understanding MPA as a complex performance-related experience rather than simply as stage fright. Papageorgi et al. (2007) proposed a conceptual framework in which anxiety develops through the interaction of the performer, the performance task, and the performance situation, and may change before, during, and after performance. This temporal and contextual understanding is useful for the present article because it shows that MPA is shaped not only by internal symptoms, but also by preparation, situational demands, self-appraisal, evaluation, and post-performance reflection.

Much of this research has also examined how individual psychological factors shape the intensity and experience of MPA. Empirical studies have linked MPA to perfectionistic concerns, trait anxiety, and broader patterns of social and performance-related fear (Kenny et al., 2004; Kobori et al., 2011; Steptoe and Fidler, 1987). Research among university music students has further found that lower self-efficacy is associated with higher levels of MPA (Hu et al., 2024; Zhang and Salarzadeh Jenatabadi, 2024). Such work has been valuable in showing that MPA is not caused only by the performance event itself, but is also shaped by performers’ expectations, self-perceptions, and concerns about evaluation. At the same time, because these studies commonly focus on individual psychological variables, they may encourage an understanding of MPA primarily as a problem located within the performer.

Taken together, psychological and performance-based perspectives have greatly advanced understanding of the personal, temporal, and situational dimensions of MPA. However, they do not always fully address how anxiety is experienced, interpreted, and responded to by students within the relationships, teaching practices, and evaluative cultures of higher music education. They therefore provide a strong foundation for this article, but not yet a complete educational account of the problem.

#### Existing approaches and their limits in higher music education

Existing approaches to MPA have offered important forms of support for musicians. Brugués (2011) reviews cognitive and behavioral strategies, relaxation-based methods, body-awareness approaches, and other forms of anxiety management. Such work has been valuable in showing that MPA is not simply something musicians must endure, but something that can be addressed in intentional ways.

However, in higher music education these approaches also reveal certain limits. Much of the existing literature continues to frame MPA primarily as a problem of treatment, coping, or symptom control. This perspective is useful, but it may also narrow the focus of support to reducing anxiety symptoms alone. In educational settings, students’ experiences of MPA are often tied not only to stress management, but also to confidence, self-understanding, musical expression, and their ongoing development as performers and learners.

This limitation becomes clearer when pedagogy is considered. Patston (2014) argues that MPA has important implications for music educators, and Clearman (2020) similarly emphasizes the need for more effective pedagogical support. However, the challenge is not only recognizing MPA as an educational issue but also translating this recognition into forms of support that can be used within everyday music teaching. Blair and van der Sluis’s (2022) systematic review highlight this problem, showing that, despite extensive research on MPA, relatively few approaches can be readily integrated into higher music education. This gap is also reflected in the experiences of both students and teachers. Tahirbegi (2022) found that students do not always disclose their experiences of MPA to teachers, while Sieger (2017) identified differences in teachers’ preparation and confidence in responding to students’ anxiety. Taken together, these studies suggest that the central problem is not simply a lack of knowledge about MPA, but the limited translation of this knowledge into consistent pedagogical and relational support within musicians’ training.

This article builds on this pedagogical gap by developing a creative arts-informed perspective on what MPA support might involve in higher music education. Rather than focusing mainly on symptom management, the proposed framework emphasizes students’ embodied awareness, expressive exploration, relational witnessing, and reflective meaning-making. In doing so, it extends existing pedagogical discussions of MPA by offering a more holistic conceptual basis for future workshop development.

### Reframing MPA beyond coping

#### MPA as an embodied and wellbeing-related experience

In higher music education, MPA should be understood not only as a performance problem, but also as an embodied and wellbeing-related experience. Building on the psychological and performance-based understandings discussed above, the perspective developed here considers how anxiety is experienced through the body and how it may shape students’ confidence, learning, wellbeing, and developing identities as musicians. Although MPA is often discussed in terms of fear, worry, or negative thinking, musicians do not experience it only at the cognitive level.

This embodied dimension is especially important for a creative arts-informed perspective. MPA is often felt through bodily sensations such as shaking, dry mouth, increased heart rate, constricted breathing, and muscular tension (Clearman, 2020). These reactions may interfere with musical control and expression, but they may also influence how students understand and relate to their bodies as performers. From the perspective developed in this article, such bodily experiences should not be treated only as symptoms to be eliminated. They may also become starting points for awareness, exploration, and reflection.

Understanding MPA in this way also highlights its connection to student wellbeing. When anxiety affects students’ confidence, self-perception, sense of musical agency, and emotional responses to performance, it becomes more than a technical obstacle to successful performance. It is also connected to how students experience themselves within the pressures, expectations, and relationships of musical learning. This provides a basis for moving beyond symptom control alone and toward more process-oriented forms of support that attend to students’ bodies, emotions, relationships, and musical lives.

#### From symptom control to process-oriented support

Many support approaches to MPA have treated anxiety as something to be reduced, controlled, or overcome. This orientation has produced useful interventions, especially for symptom relief and coping in performance contexts. However, Kenny’s (2005) review found that the limited number, small samples, and methodological quality of existing treatment studies made it difficult to draw firm conclusions about their effectiveness. This suggests that a support model based only on elimination or control may be too limited, particularly in higher music education where anxiety is often woven into students’ learning, self-evaluation, and musical development.

From an educational perspective, the central issue is not whether anxiety can be completely removed, but how students can be supported to relate to it differently. Acceptance-based approaches offer an important starting point for this shift. Juncos et al. (2017) frame Acceptance and Commitment Therapy for MPA as aiming not at direct symptom reduction, but at increasing psychological flexibility in relation to anxious thoughts and feelings. Clarke et al. (2020) similarly report that an ACT-based group intervention for student vocalists focused on increasing flexibility and reducing inflexibility, rather than requiring anxiety itself to disappear. These studies are valuable because they challenge the assumption that successful support must mean the absence of anxiety.

Students may need opportunities not only to accept anxiety cognitively, but also to explore it through embodied, creative, relational, and meaning-making processes within music education itself. This process-oriented perspective is consistent with Rogers’ person-centred expressive arts approach, in which creative activity is valued primarily as a means of self-exploration, self-expression, and access to personal meaning, rather than for the aesthetic quality of the final product (Rogers, 1993; Estrella, 1997). In this way, support can move from symptom reduction alone toward awareness, acceptance, self-regulation, flexibility, and adaptive engagement. Rather than treating anxiety only as evidence of failure, students may be supported to notice it, make sense of it, regulate their responses, and remain connected to musical action. This orientation is particularly relevant in educational contexts because MPA support should address not only immediate performance outcomes, but also students’ longer-term growth, self-understanding, and sustainable artistic development.

#### The relevance of creative arts-informed thinking

If support for MPA is not limited to symptom reduction, then creative arts-informed thinking becomes especially relevant. In this article, creative arts-informed support is understood in educational rather than clinical terms. It does not mean transferring art therapy, music therapy, or dance movement therapy directly into higher music education. Instead, it draws on creative, embodied, and expressive processes to help students notice, express, externalize, share, and reinterpret anxiety within a pedagogical setting.

Visual art making has been associated with reductions in cortisol, suggesting a possible stress-related physiological effect (Kaimal et al., 2016). Research on dance and dance movement therapy has also reported positive health-related psychological outcomes (Koch et al., 2019). More broadly, reviews of arts and health research suggest that music, visual art, movement, writing, and other creative practices may support emotional expression and wellbeing across a range of contexts (Hu et al., 2021; Stuckey and Nobel, 2010). Although much of this evidence comes from clinical or health-related contexts, it helps explain why artistic and embodied activity may be relevant to MPA when adapted carefully within an educational frame.

This provides the conceptual bridge from existing MPA literature to the creative arts perspective proposed in this article. Psychological and performance-based research has helped explain what MPA is and how it functions, while acceptance-based work has shown the value of changing one’s relationship with anxiety rather than simply trying to eliminate it. Creative arts-informed thinking extends these insights by considering how students might encounter anxiety in more embodied, expressive, relational, and meaning-making ways within music education itself.

### The proposed framework: a creative arts perspective on MPA support

#### A conceptual framework for MPA support

Building on this reframing, the central contribution of this article is the four-part framework conceptually synthesized here. MPA is understood not merely as clinical symptoms to be reduced, but as an embodied, emotional, relational, and meaning-laden experience situated within the culture of music learning. This framework identifies four interrelated pedagogical processes through which anxiety can be explored within higher music education: embodied awareness, expressive exploration, relational witnessing, and reflective meaning-making.

Embodied awareness refers to helping students notice how anxiety is felt through breath, posture, tension, movement, and bodily readiness. Expressive exploration offers non-verbal and symbolic ways to externalize and reshape anxiety through sound, movement, drawing, writing, or improvisation. Relational witnessing creates opportunities for students to experience being seen and heard in a less judgmental context, which is especially important in performance cultures shaped by evaluation, comparison, and fear of mistakes. Reflective meaning-making supports students in reconsidering what anxiety, performance, musical success, and artistic identity mean to them.

These four processes are not presented as separate techniques, but as connected dimensions of a creative arts perspective on MPA support. Together, they translate the broader argument of this article into a more specific educational structure. Rather than presenting a finished intervention model, the framework identifies the key pedagogical processes through which future non-clinical MPA support might be developed. In this way, it provides a conceptual bridge between existing MPA research and the design of creative arts-informed workshops in higher music education.

#### From conceptual framework to future workshop development

A further implication of this framework is the possible development of embodied and creative arts-informed workshop models in higher music education. If MPA is understood as more than a symptom to be reduced, and as something experienced through the body, emotion, reflection, relationship, and performance, then workshop-based approaches may offer a useful format for educational support.

Such workshops may be valuable because they allow support to move beyond verbal explanation alone. Through activities such as movement, music, improvisation, sound, imagery, writing, and multimodal reflection, students may explore anxiety in ways that are experiential as well as cognitive. Workshop contexts may therefore help students develop greater awareness of how anxiety is experienced, expressed, and regulated in and through the body, while also providing opportunities for shared reflection and supportive witnessing.

At the same time, future workshop development would require careful ethical and pedagogical attention. In higher music education, its value would lie in providing a structured, safe, and non-clinical educational process through which students can explore and respond to MPA as part of musical learning. To ensure student safety and professional boundaries, future workshop protocols must incorporate clear screening and referral mechanisms; this ensures that participants experiencing severe or clinical-level anxiety are safely identified and seamlessly directed to university counseling services or specialized psychological support. Future research should therefore examine how such workshops can be designed, facilitated, and safely evaluated within these clear pedagogical and ethical parameters in specific higher music education contexts.

## Discussion

The central contribution of this article is to synthesize MPA research, higher music education scholarship, and creative arts-informed thinking into a broader educational perspective on performance anxiety. Building on psychological and performance-based research that has clarified the multidimensional nature of MPA, the article proposes a creative arts perspective organized around four interrelated pedagogical processes: embodied awareness, expressive exploration, relational witnessing, and reflective meaning-making. Through this framework, MPA support is understood not only in terms of symptom reduction, but also as a process through which students may explore the embodied, emotional, relational, and meaning-making dimensions of anxiety within musical learning.

This perspective extends existing discussions of MPA by positioning performance anxiety as both an individual experience and a pedagogical and wellbeing-related concern in higher music education. It does not replace psychological or clinical approaches, which remain essential for students requiring specialist support. Instead, it expands the educational possibilities for MPA support by considering how students might engage with anxiety through creative, reflective, and relational processes. This is particularly relevant in higher music education, where MPA is closely connected to assessment, artistic identity, teacher-student relationships, peer comparison, and students’ developing sense of themselves as musicians. Future research should examine how this framework can be translated into creative arts-informed workshops, how students experience such support, and what pedagogical, ethical, and institutional conditions are needed for its responsible use in higher music education.

## Data Availability

The original contributions presented in the study are included in the article/supplementary material, further inquiries can be directed to the corresponding author.
